# A Novel Orthotopic Patient-Derived Xenograft Model of Radiation-Induced Glioma Following Medulloblastoma

**DOI:** 10.3390/cancers12102937

**Published:** 2020-10-12

**Authors:** Jacqueline P. Whitehouse, Meegan Howlett, Hilary Hii, Chelsea Mayoh, Marie Wong, Paulette Barahona, Pamela Ajuyah, Christine L. White, Molly K. Buntine, Jason M. Dyke, Sharon Lee, Santosh Valvi, Jason Stanley, Clara Andradas, Brooke Carline, Mani Kuchibhotla, Paul G. Ekert, Mark J. Cowley, Nicholas G. Gottardo, Raelene Endersby

**Affiliations:** 1 Brain Tumour Research Program, Telethon Kids Institute, Nedlands 6009, Australia; Jacqueline.Whitehouse@telethonkids.org.au (J.P.W.); Meegan.Howlett@telethonkids.org.au (M.H.); Hilary.Hii@telethonkids.org.au (H.H.); Santosh.Valvi@health.wa.gov.au (S.V.); jason.stanley2997@gmail.com (J.S.); Clara.Andradas@telethonkids.org.au (C.A.); Brooke.Carline@telethonkids.org.au (B.C.); Mani.Kuchibhotla@telethonkids.org.au (M.K.); Nick.Gottardo@health.wa.gov.au (N.G.G.); 2 Centre for Child Health Research, University of Western Australia, Nedlands 6009, Australia; 3 Children’s Cancer Institute, Lowy Cancer Centre, UNSW Sydney, Kensington 2033, Australia; CMayoh@ccia.org.au (C.M.); MWong-Erasmus@ccia.org.au (M.W.); pbarahona@ccia.org.au (P.B.); pajuyah@ccia.org.au (P.A.); PEkert@ccia.org.au (P.G.E.); MCowley@ccia.org.au (M.J.C.); 4 School of Women’s and Children’s Health, UNSW Sydney, Kensington 2033, Australia; 5 Genetics and Molecular Pathology Laboratory, Hudson Institute of Medical Research, Clayton 3168, Victoria, Australia; christine.white@hudson.org.au (C.L.W.); molly.buntine@hudson.org.au (M.K.B.); 6 Department of Molecular and Translational Science, Monash University, Melbourne 3168, Victoria, Australia; 7 Department of Neuropathology, PathWest Laboratory Medicine, Royal Perth Hospital, Perth 6000, Australia; Jason.Dyke@health.wa.gov.au; 8 Pathology and Laboratory Medicine, University of Western Australia, Nedlands 6009, Australia; 9 Department of Neurosurgery, Perth Children’s Hospital, Nedlands 6009, Australia; Sharon.Lee@health.wa.gov.au; 10 Department of Paediatric and Adolescent Oncology/Haematology, Perth Children’s Hospital, Nedlands 6009, Australia; 11 Division of Paediatrics, University of Western Australia Medical School, Nedlands 6009, Australia; 12 Murdoch Children’s Research Institute, Royal Children’s Hospital, Parkville 3052, Victoria, Australia; 13 Peter MacCallum Cancer Centre, Melbourne 3000, Victoria, Australia

**Keywords:** diffuse midline glioma, radiation-induced glioma, diffuse intrinsic pontine glioma, patient-derived xenograft, medulloblastoma, radiation, paediatric cancer, brain cancer

## Abstract

**Simple Summary:**

Radiation-induced glioma (RIG) is a highly aggressive brain cancer arising as a consequence of radiation therapy, for which there is currently no effective treatment. In order to test new drugs in the hope of finding more effective therapies, we need mouse models that faithfully replicate human RIG. Our laboratory collected tumour cells at autopsy from a paediatric patient with RIG following treatment for a different brain tumour. Using these cells, we created a mouse brain tumour model that retains all the characteristics and features of the original patient tumour from which it was derived. This unique model allowed us to study the progression of RIG in the brain, and to identify drugs that may be effective in the treatment of this disease. This mouse model will also allow us to test the efficacy of new treatments, with the hope of improving the prognosis for patients diagnosed with this disease.

**Abstract:**

Radiation-induced glioma (RIG) is a highly aggressive brain cancer arising as a consequence of radiation therapy. We report a case of RIG that arose in the brain stem following treatment for paediatric medulloblastoma, and the development and characterisation of a matched orthotopic patient-derived xenograft (PDX) model (TK-RIG915). Patient and PDX tumours were analysed using DNA methylation profiling, whole genome sequencing (WGS) and RNA sequencing. While initially thought to be a diffuse intrinsic pontine glioma (DIPG) based on disease location, results from methylation profiling and WGS were not consistent with this diagnosis. Furthermore, clustering analyses based on RNA expression suggested the tumours were distinct from primary DIPG. Additional gene expression analysis demonstrated concordance with a published RIG expression profile. Multiple genetic alterations that enhance PI3K/AKT and Ras/Raf/MEK/ERK signalling were discovered in TK-RIG915 including an activating mutation in *PIK3CA*, upregulation of *PDGFRA* and *AKT2*, inactivating mutations in *NF1*, and a gain-of-function mutation in *PTPN11*. Additionally, deletion of *CDKN2A/B*, increased *IDH1* expression, and decreased *ARID1A* expression were observed. Detection of phosphorylated S6, 4EBP1 and ERK via immunohistochemistry confirmed PI3K pathway and ERK activation. Here, we report one of the first PDX models for RIG, which recapitulates the patient disease and is molecularly distinct from primary brain stem glioma. Genetic interrogation of this model has enabled the identification of potential therapeutic vulnerabilities in this currently incurable disease.

## 1. Introduction

Tumours arising in the brain stem, including diffuse midline glioma (DMG) with histone H3 K27M mutation, often referred to as diffuse intrinsic pontine glioma (DIPG), are among the most aggressive types of paediatric brain cancer. Recent molecular characterisation of paediatric high grade gliomas [1,2,3] led to the World Health Organisation (WHO) redefining central nervous system (CNS) diagnostic criteria and diffuse gliomas (including DIPG) were reclassified into more precise entities including ‘DMG, H3 K27M-mutant’, ‘Glioblastoma (GBM), IDH1-mutant’ and ‘GBM, IDH1-wildtype’ [4,5]. Despite these new diagnostic entities there are still no effective treatments for brain stem glioma and most children with this disease will pass away within a year of diagnosis, with only 2.6% of patients surviving more than five years [6]. Gliomas can occur as primary brain tumours but may also arise as a consequence of radiation therapy in survivors of other paediatric brain tumours, such as medulloblastoma or ependymoma. Radiation-induced gliomas (RIGs) are at least as, if not more, aggressive than spontaneously occurring gliomas [7,8,9], and are invariably fatal, with a cumulative incidence ranging from 0.3% to 3.96% following radiation treatment for paediatric brain tumours [8,10].

A small study of five RIGs reported that these tumours are histologically and cytogenetically indistinguishable from de novo paediatric high-grade gliomas [7]. Indeed, the current clinical standard of care is identical for these two tumour types. There is developing evidence that the mutational and gene expression profiles of paediatric RIGs are distinct from their primary counterparts, suggesting clinical care may need to reflect this. Unfortunately, extensive investigations into the genetic alterations that define paediatric RIGs are limited. This is partly due to the rarity of this tumour type and the paucity of biological material available. Understandably, biopsies have been particularly rare for RIGs located within the critical brain stem region in favour of a radiographic diagnosis [11], although this may be slowly changing [12,13]. RIGs have also been mistakenly diagnosed as relapses of the primary tumour [14,15], further reducing the availability of correctly identified RIG samples for analysis.

Studies investigating molecular alterations in paediatric RIGs are few and report small numbers of patient samples. These studies suggest that paediatric RIGs demonstrate more overlap with pilocytic astrocytomas (PAs) [7] and adult primary GBMs [8] than with paediatric high-grade gliomas. Overexpression of *ERBB3, SOX10* and *PDGFRA* has been reported in paediatric RIG [7,14], as well as mutations in *TP53*, *PDGFRA* and *PIK3CA*, homozygous deletion of *CDKN2A* and *MTAP*, and alterations in multiple receptor tyrosine kinase and Ras-Raf-MAPK genes [8,14,16,17,18,19,20]. Of note, RIGs lack genetic alterations characteristic of major diffuse glioma subtypes including mutations in *IDH1, IDH2, H3F3A, HIST1H3B HIST1H3C, ACVR1* or *TERT* [8,14,18,21,22], although *EGFR* amplifications and mutations or deletions in *PTEN* have been reported in a small number of cases [7,8,14,16,18].

The limited molecular data available precludes any real ability of researchers and clinicians to definitively characterise and understand these tumours. In order to discover more effective treatments and to improve survival rates for these patients, we need models through which we can identify potential molecular targets and then test appropriate therapeutics preclinically. Whilst in vitro models are useful, there is a limit to their translational utility, and indeed variation in culture methods can have a significant impact on gene expression and drug responses [23]. The most robust, efficient and effective models are orthotopic patient-derived xenograft (PDX) models.

PDX models are the gold-standard in cancer research for understanding disease progression and for preclinical testing of new therapies. Although they can be challenging to establish, PDX models reward researchers by closely recapitulating the heterogeneity of different individual cancers [24,25], particularly in comparison to genetically-engineered models of human cancer that rely on a small number of significant genetic alterations to drive tumour development [26]. Once established, rigorous molecular characterisation of PDX models is essential to understand in detail the disease that the PDX represents. The only reported PDX mouse models of RIG were very recently published and were derived from biopsy and autopsy tissue samples from a single patient [15]. Here, we describe and characterise a PDX model of a RIG following treatment for medulloblastoma, which faithfully recapitulates the patient disease and is molecularly distinct from primary DMG.

## 2. Results

### 2.1. Case Report

**Figure 1:**
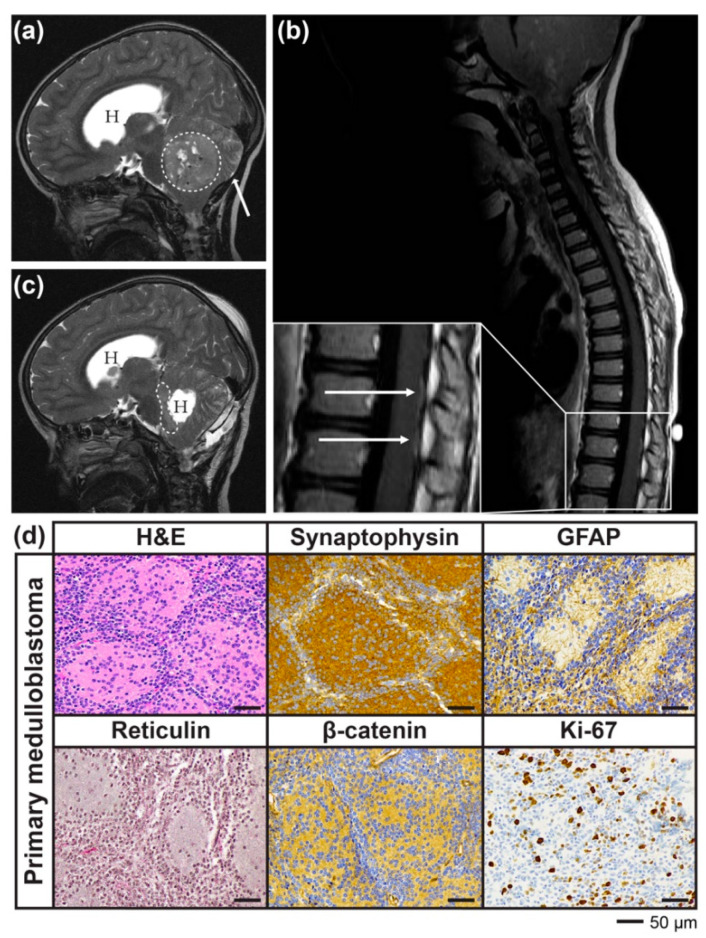
Diagnostic and post-operative magnetic resonance imaging (MRI) and histological characterisation of the primary medulloblastoma. (**a**) Diagnostic cranial MRI depicting posterior fossa mass (dashed line) and accompanying hydrocephalus (H). White arrow indicates leptomeningeal spread. (**b**) Spinal MRI revealed drop metastases in distal spinal cord. Inset: arrows indicate metastatic lesions. (**c**) Post-operative MRI showed residual disease within the left cerebello-pontine angle (dashed line) and hydrocephalus (H). (**d**) Histological staining of patient cerebellar tumour tissue. Haematoxylin and eosin (H&E) shows small, round, blue cells arranged in a compact and nodular fashion. Tumour cells were positive for synaptophysin on a background of glial fibrillary acidic protein (GFAP)-positive astrocytic processes. The tumour cells were negative for reticulin and showed only cytoplasmic staining for β-catenin, consistent with a non-SHH, non-WNT medulloblastoma. Ki-67 proliferative index was 30%.

A previously well four-year-old male presented with a one-week history of vomiting and headache. Magnetic resonance imaging (MRI) of the brain revealed the presence of a large posterior fossa mass with accompanying hydrocephalus (Figure 1a). There was evidence of leptomeningeal spread over the surface of the cerebellum and drop metastases in the distal spinal cord (Figure 1a,b). There was no history of cancer in other family members.

The patient underwent resection of the majority of the mass; however, residual disease was evident within the left cerebello-pontine angle on the post-operative MRI (Figure 1c). Histopathology reporting at the time recorded a nodular medulloblastoma; however, independent pathological review since reclassified this tumour histologically as classic medulloblastoma and molecularly as Group 4 (case 6 in [8]). Haematoxylin and eosin (H&E) staining showed a tumour composed of small, round, blue cells with both compact and nodular architecture. The malignant cells were positive for neuronal marker synaptophysin and negative for glial marker glial fibrillary acidic protein (GFAP), which stained background astrocytic processes only. Absence of reticulin staining, and β-catenin staining confined to tumour cell cytoplasm was in keeping with a non-SHH, non-WNT medulloblastoma subtype. The proliferation index as measured by Ki-67 was approximately 30% (Figure 1d).

The patient was treated as per the Children’s Oncology Group (COG) protocol CCG-99701 Arm B, with radiation and chemotherapy [27]. Radiation dose was 36 Gy to the craniospinal axis with boost doses to the posterior fossa of 19.8 Gy, to the optic chiasm of 14.4 Gy, to the lower thoracic spine up to the sacrum of 9 Gy and to the cauda equina of 14.4 Gy. Carboplatin and vincristine were used as radiosensitising agents. The patient further received six cycles of chemotherapy consisting of cyclophosphamide, cisplatin and vincristine. An MRI performed at the end of therapy nine months later showed no evidence of residual or recurrent disease. The patient was followed up regularly with clinical examination and MRI scans. Late effects of therapy included neuro-cognitive impairment, bilateral hearing loss, panhypopituitarism, bilateral cataracts and scoliosis/kyphosis, which were appropriately managed.

**Figure 2:**
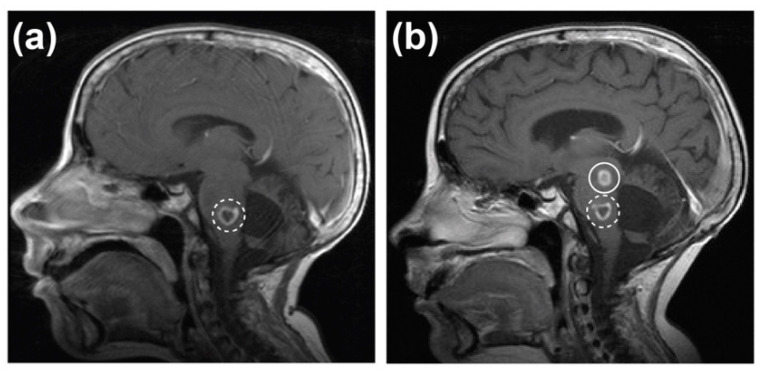
Magnetic Resonance Imaging (MRI) indicated midline glioma and fungal lesions within the brain. (**a**) MRI performed 11 years after initial medulloblastoma diagnosis depicting mass within the pons and upper medulla (dashed line). (**b**) MRI performed three months after initial diffuse intrinsic pontine glioma diagnosis depicting midline glioma (dashed line) and one of multiple suspected fungal lesions (solid line).

At nearly 16 years of age, and 11 years after the initial diagnosis of medulloblastoma, the patient presented with a two-week history of left hand and left foot numbness. Clinical examination revealed a new left-sided seventh cranial nerve palsy and mild left upper limb weakness with reduced sensations in a glove and stocking distribution. An MRI showed an enlarging mass within the pons and upper medulla with an expanding lesion showing an area of cavitation and rim enhancement at its posteroinferior margin (Figure 2a). These imaging characteristics were suggestive of a high-grade glioma rather than relapsed metastatic medulloblastoma. As a biopsy of this lesion was considered inappropriate with the risks involved, a diagnosis of DIPG was delivered based on clinical and radiological features alone. He commenced treatment as per the COG clinical trial ACNS0927 [28] and received 54 Gy of focal radiation therapy with vorinostat. He showed some clinical improvement with reduction in the size of the pontine lesion after radiation; however, within three months multiple punctate and ring-enhancing lesions developed in the supra- and infratentorial regions of the brain (Figure 2b). A biopsy of one of the lesions indicated inflammatory changes but could not determine the cause of the lesion. There were also multiple bilateral lung nodules present and a lung biopsy performed at the same time indicated a fungal infection. This suggested that the lesions in the brain were likely to be fungal in nature secondary to his immunocompromised state from steroids and vorinostat, and this was later confirmed at autopsy. The patient was treated with voriconazole; however, during this period there was further progression of the glioma and he passed away eight months after the diagnosis of DIPG.

### 2.2. Development of a PDX Model of RIG Following Medulloblastoma that Histologically Recapitulates the Patient Tumour

**Figure 3:**
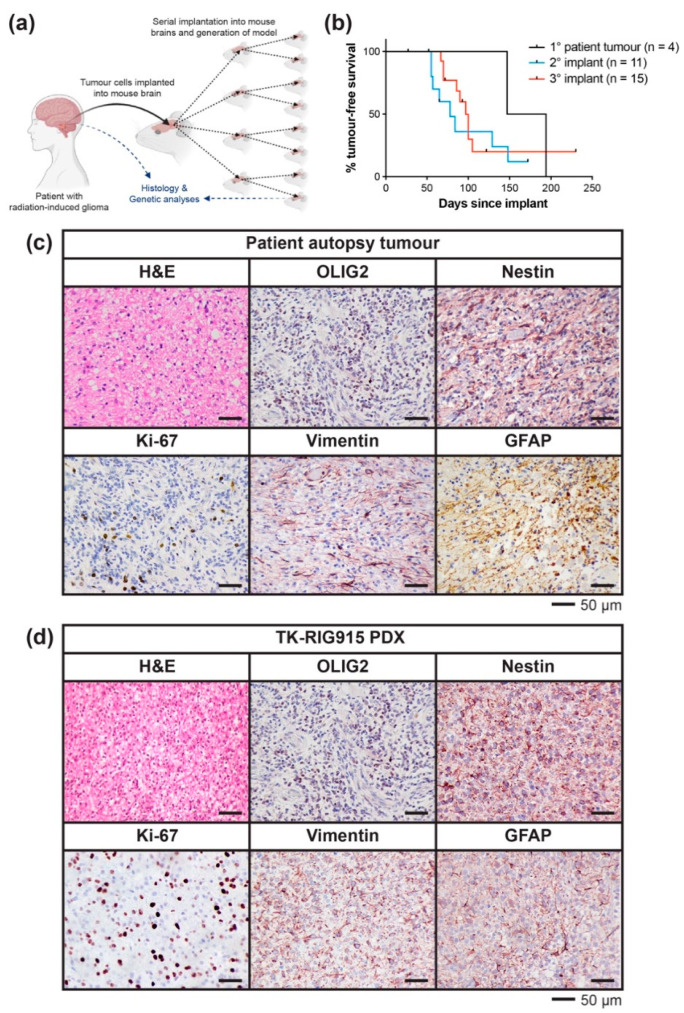
Development, survival characteristics and histological features of TK-RIG915. (**a**) Schematic depicting the generation of TK-RIG915. Where possible, tumour tissue was stored for genetic analyses. (**b**) Time to morbidity in the TK-RIG915 patient derived-xenograft (PDX) model for mice implanted with patient tumour cells (black), or serially transplanted with PDX tumour cells (secondary implant, blue; tertiary implant, red). Subjects euthanised due to non-tumour-related reasons were censored (vertical black dash). Histological assessment of (**c**) patient tumour tissue obtained at autopsy and (**d**) tumour tissue from TK-RIG915 PDX demonstrates that the PDX recapitulates the patient tumour histologically. Haematoxylin and eosin (H&E) staining depicts a highly cellular tumour and immunohistochemistry (IHC) for Ki-67 confirms active proliferation. Positivity for OLIG2, nestin, vimentin and glial fibrillary acidic protein (GFAP) by IHC support a glial phenotype.

A PDX model (TK-RIG915) of this disease was successfully generated by implanting tumour cells isolated post-mortem into the brains of immunodeficient mice. Upon tumour-related morbidity, tumours were removed, dissociated and reimplanted into the brains of successive recipient animals (Figure 3a). Initially, mice implanted with the patient cells had a median time to morbidity of approximately 170 days, which reduced to approximately 78 days and 100 days in the secondary and tertiary implant generations, respectively (Figure 3b). Whilst 100% of mice implanted with the patient tumour cells developed tumour-related morbidities, tumour penetrance of successive generations was difficult to accurately calculate due to a significant number of immunocompromised recipient mice requiring euthanasia due to non-tumour-related complications common to this strain, including rectal prolapse or serious skin conditions (Table S1). Once established, we confirmed that the PDX tumour was derived from the matched patient tumour via Short Tandem Repeat (STR) analysis (Table S2).

Histological assessment of the patient tumour and matched PDX tumours was performed in order to characterise the model and ensure that the PDX faithfully recapitulated the original patient tumour (Figure 3c,d). H&E staining showed a highly cellular neoplasm composed of compact cells with oval nuclei, moderate nuclear pleomorphism and predominantly fibrillary cytoplasm. Both the patient and the PDX tumours were positive for OLIG2, nestin, vimentin and GFAP, with focal Ki-67 proliferative indices of up to 25% in the patient tumour and 40% in PDX tumours, respectively. This staining pattern was consistent with a glial phenotype and demonstrated that the PDX faithfully recapitulated the patient tumour histologically.

### 2.3. TK-RIG915 is Molecularly Distinct from Primary DMG and Matches a RIG Expression Profile

We sought to fully molecularly characterise TK-RIG915 and the matched patient tumour by performing methylation profiling, RNA sequencing (RNAseq), and whole genome sequencing (WGS) analysis. Using methylation arrays, we attempted to determine the best-fit tumour subclass using the Molecular Neuropathology (MNP) 2.0 classifier [29]. Neither the patient nor the PDX tumours successfully classified (calibrated score ≥ 0.9) with one of the > 80 known tumour subclasses in the MNP database, including ‘DMG with H3 K27M mutation’. The patient tumour sample best matched the class “Control tissue, inflammatory tumour microenvironment” with a calibrated score of 0.63. The PDX sample had no matching scores above 0.3. Whilst the brain stem tumour did not classify as the primary diagnosis of Group 4 medulloblastoma, the MNP2.0 classifier failed to identify which tumour type it was. It is important to note that a RIG subgroup does not currently exist in the MNP2.0 classifier. The finding that this tumour was not DMG supports a previous report where whole exome sequencing suggested this patient’s tumour may be molecularly distinct from primary DMG (case 6 in [8]).

**Figure 4:**
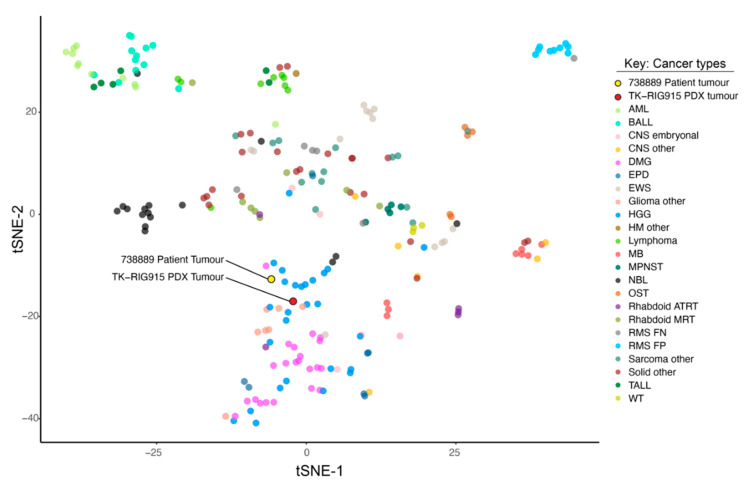
TK-RIG915 and the matched patient tumours do not cluster with diffuse midline gliomas (DMGs) by RNA expression. T-distributed Stochastic Neighbour Embedding (tSNE) analysis was performed using RNA sequencing data from ZERO with patient primary sample (labelled 738,889 Patient tumour) and patient-derived xenograft (PDX) sample (labelled TK-RIG915 PDX tumour). tSNE-1 and tSNE-2 refer to the first two dimensions of the tSNE. ZERO cohort consists of high-risk paediatric tumours. Legend: AML—acute myeloid leukaemia, BALL—B-cell acute lymphoblastic leukaemia, DMG—Diffuse Midline Glioma, EPD—Ependymoma, EWS—Ewing Sarcoma, HGG—High-Grade Glioma, HM other—other Haematological Malignancies, MB—Medulloblastoma, MPNST, Malignant Peripheral Nerve Sheath Tumour, NBL—Neuroblastoma, OST—Osteosarcoma, Rhabdoid ATRT—Rhabdoid Atypical Teratoid Rhabdoid Tumour, Rhabdoid MRT—Rhabdoid Malignant Rhabdoid Tumour, RMS FN—Rhabdomyosarcoma Fusion Negative, RMS FP—Rhabdomyosarcoma Fusion Positive, TALL—T-cell acute lymphoblastic leukaemia, WT—Wilms Tumour.

In the absence of a clear CNS tumour classification based on methylation profiling, we performed clustering analysis using RNA expression data to determine which brain tumour subtype our samples most closely related to. We compared the expression profile of the patient and the PDX model against a cohort obtained through the ZERO childhood cancer precision medicine program of high-risk paediatric cancers (ZERO, *n* = 229) [30]. Both the patient and the PDX tumours clustered close to each other, highlighting retention of the molecular characteristics of the patient tumour in the PDX model (Figure 4). The PDX and matched patient tumours clustered most closely with high grade gliomas and other gliomas in the reference cohort, concordant with the nature of these tumours. Of note, the patient and PDX samples did not cluster with other DMG samples in this cohort, again supporting previous findings that this case is distinct from primary DMG [7,8].

**Figure 5:**
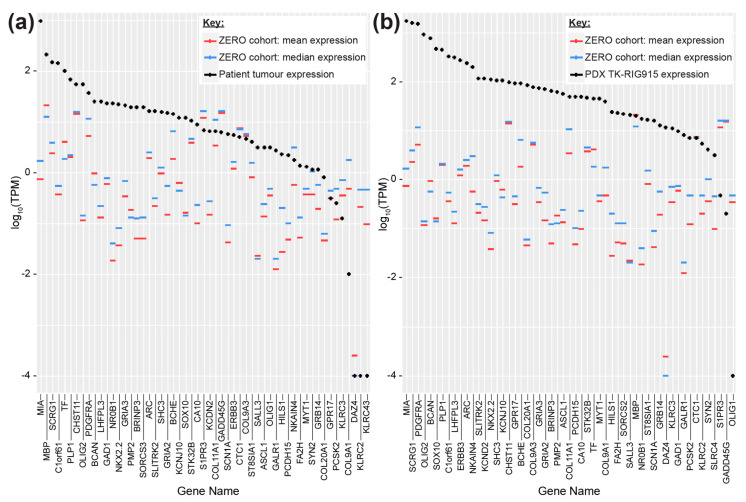
Gene expression in the patient tumour tissue and TK-RIG915 correlated with a radiation-induced glioma (RIG) expression profile. Transcript abundance from RNAseq data (y axis, log 10 scaled transcripts per million, TPM) for genes reported to be upregulated in RIG [7] (x axis) were determined from (**a**) patient and (**b**) TK-RIG915 tumours (black). By way of comparison, the mean (red) and median (blue) transcript abundance for these genes was lower in the ZERO reference cohort consisting of various high-risk paediatric tumours indicating less concordance with the RIG expression profile for other paediatric cancers.

We compared the patient’s clinical history with the criteria defined by Cahan et al. [31], which is currently being used as the standard to define radiation-induced malignancies [32]. The patient had no genetic predisposition for the development of secondary tumours, and the brainstem tumour arose within the irradiated field, occurred more than four years after the delivery of radiation, and was histologically distinct from the primary medulloblastoma, thereby satisfying all criteria to be considered a radiation-induced secondary tumour. Given this clinical history, we compared the gene expression of the patient and PDX samples to a previously published, independent subset of genes reported to be overexpressed in RIGs [7]. High expression levels of the majority of the RIG-associated genes were observed in both the patient and PDX tumours in comparison to the reference cohort, supporting the finding that this case and the TK-RIG915 PDX model are mostly likely RIGs (Figure 5).

### 2.4. TK-RIG915 Genetically Recapitulates the Original Patient Tumour and Does Not Harbour a Number of Key Glioma-Associated Genetic Alterations

At the time of diagnosis, the patient tumour from which TK-RIG915 was derived was diagnosed and treated as DIPG; however, our analysis of DNA methylation and RNA expression suggested that this disease, and the subsequent derived PDX model, is a RIG and molecularly distinct from primary DIPG. Therefore, we sought to characterise the molecular mechanisms driving tumour growth in TK-RIG915 by interrogating the DNA methylation and RNAseq data further, as well as performing WGS analysis on the tumour and matched germline DNA from this patient.

**Figure 6:**
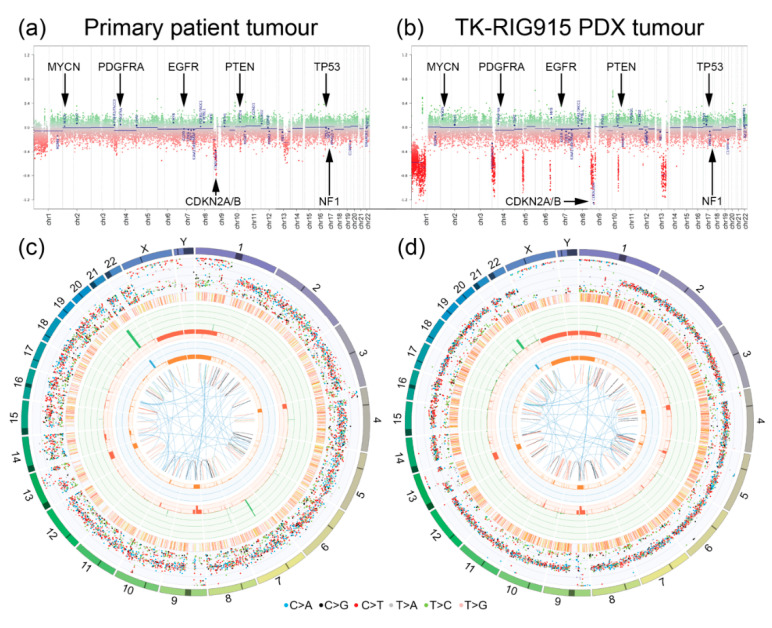
Genomic DNA analyses demonstrate concordance between the patient tumour and patient-derived xenograft (PDX) TK-RIG915. (**a**,**b**) Copy number variant plots for (**a**) the patient tumour and (**b**) the matched PDX TK-RIG915 tumour depicting loss of *CDKN2A/B* and hemizygous loss of chromosome 1p. Selected glioma-associated genes are indicated. (**c**,**d**) CIRCOS plots for (**c**) the patient tumour and (**d**) the PDX TK-RIG915 tumour show the range of molecular alterations observed in these tumours. Key to the CIRCOS plots: Outermost circle indicates the chromosomes, where darker shading represents large gaps in the human reference genome (e.g., centromeres). Second circle (grey shading) shows the somatic variants. These are divided into an outer ring of single nucleotide variants where each dot represents a single variant coloured as shown with allele frequencies (corrected for tumour purity and scaled from 0–100%) and an inner ring of short insertions and deletions (yellow and red, respectively). Third circle (red and green shading) shows all observed tumour purity-adjusted copy number changes (losses and gains indicated in red and green, respectively, scale ranges from 0 (complete loss to 6 (high level gains)). Fourth circle (orange and blue shading) represents the observed ‘minor allele copy numbers’ across the chromosome, ranging from 0 to 3. The expected normal minor allele copy number is 1. Values below 1 are shown as a loss (orange) and represents a loss of heterozygosity event, whilst values above 1 (blue) indicate amplification events of both alleles at the indicated locations. Innermost circle displays the observed structural variants within or between the chromosomes. Translocations are indicated in blue, deletions in red, insertions in yellow, tandem duplications in green and inversions in black.

Copy number analysis (using methylation array data [29] and WGS) of both the patient and PDX tumours revealed hemizygous loss of chromosome 1p and homozygous deletions on chromosome 9p including a segment containing *CDKN2A/B, MTAP* and multiple interferon genes (Figure 6a–d). WGS revealed that no pathogenic or likely pathogenic single nucleotide variants affecting childhood cancer predisposition genes were present in the germline and no clinically reportable somatic fusions were found. CIRCOS plots generated from the WGS data visually demonstrate the overarching similarities between the genetic profiles of the patient tumour and TK-RIG915 (Figure 6c,d and Figures S1 and S2). The variant allele frequency for somatic variants (second circle) was more varied in the patient tumour data compared to TK-RIG915, reflecting contaminating normal DNA due to the diffuse nature of this glioma (52% tumour, 48% non-tumour brain tissue) compared to TK-RIG915 (100% tumour purity after removal of mouse-specific reads during analysis).

**Figure 7:**
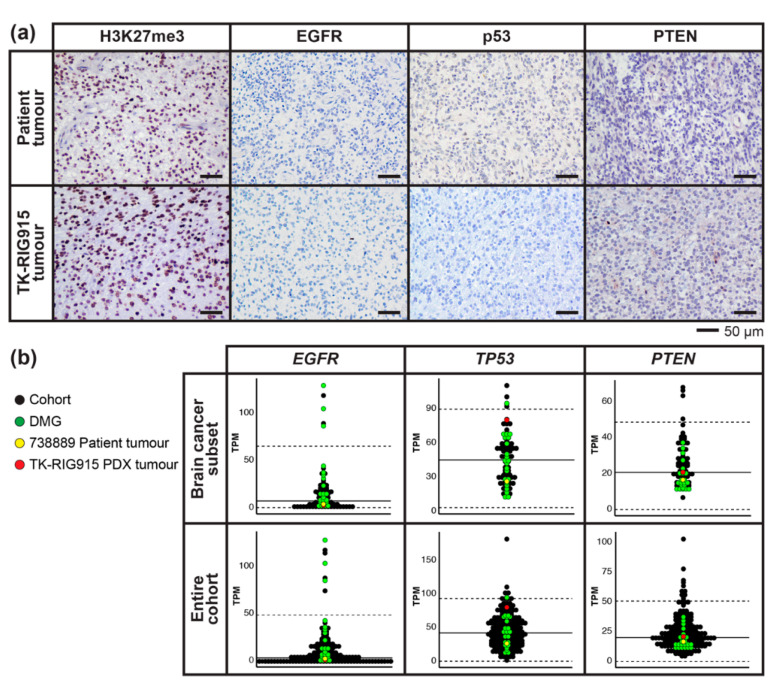
Immunohistochemistry (IHC) and RNA expression levels of glioma-associated genes in the patient tumour and matched TK-RIG915 patient-derived xenograft (PDX). (**a**) Both the patient and matched TK-RIG915 PDX tumour tissues were wildtype for Histone H3, supported by positive H3K27me3 staining. IHC for EGFR and p53 was negative, and tumours were weakly PTEN positive. Scale bar represents 50 µm. (**b**) Gene expression levels (y axis: transcripts per million, TPM) for the indicated genes. The patient tumour (yellow) and TK-RIG915 (red) are compared with two subsets of a reference cohort containing only high-risk paediatric brain tumours (top panel) or all high-risk paediatric tumours (bottom panel). Diffuse midline gliomas (DMGs) from the reference cohort are indicated in green, all other cancers are black. Solid black line shows the mean TPM of the reference cohort, and dashed line shows the TPM values that are two standard deviations away from the mean. TK-RIG915 exhibits low transcript levels of EGFR, which matches protein expression by IHC in (**a**) and unremarkable transcript levels of TP53 and PTEN, concordant with whole genome sequencing and IHC.

Of note, no mutations in histone H3 were observed. Immunohistochemistry (IHC) for H3 K27 tri-methylation (H3K27me3) confirmed this, with both the patient and PDX tumours staining positively (Figure 7a), supporting that they did not harbour the characteristic K27M variant observed in the majority of paediatric DIPGs [33,34]. Given that these cases clustered more closely with other high-grade gliomas upon tSNE analysis, and that paediatric RIGs share common mutational events with adult GBM [8], we investigated genes commonly mutated in adult high-grade glioma, namely *EGFR, TP53* and *PTEN* [35,36]. *EGFR* was not amplified (Figure 6a,b), and no mutations or deletions were found in either the patient or PDX tumours by WGS. EGFR expression was undetectable using IHC and transcript levels were very low in both cases compared to the reference datasets (Figure 7). Copy number profiling indicated that *TP53* and *PTEN* were not deleted in either the patient tumour or the PDX tumours (Figure 6a,b), and mutations in these genes were not observed by WGS. RNA expression analysis demonstrated unremarkable levels of *TP53* and *PTEN* transcripts relative to both the reference subset of high-risk paediatric brain tumours and the reference set of all high-risk paediatric tumours (Figure 7b). These data were further supported by negative staining for p53 by IHC and weakly positive staining for PTEN typical of astrocytic tumours (Figure 7a).

### 2.5. Mutations that Activate PI3K/AKT and Ras/Raf/MEK/ERK Signalling Are Present in TK-RIG915

A number of mutations known to drive PI3K/AKT and Ras/Raf/MEK/ERK signalling were observed in both the patient tumour and TK-RIG915. A known pathogenic activating mutation in *PIK3CA* [37] was observed (H1047L, Table 1). Missense mutations at this hotspot location of the catalytic subunit (p110) of PI3K have been reported in a number of cancer types [38,39,40] and result in increased kinase activity [38,41]. This finding is concordant with one of ten mutations previously reported for this case [8]. The remaining nine mutations reported by Gits et al. [8] were either found to be present in the germline DNA, or not detected in our sample (Table S3).

Pathogenic mutations in two key genes that drive the Ras/Raf/MEK/ERK pathway were identified in both the patient and PDX tumours. Neurofibromin (NF; encoded by the *NF1* gene) negatively regulates cellular proliferation by downregulating RAS activity [42]. *NF1* was found to harbour a pathogenic stop-gain truncation mutation in exon 26 (p.Glu1123Ter) as well as a frameshift mutation in exon 3 (p.Asn78LysfsTer29), neither of which were previously reported from exome sequencing [8]. Both mutations are expected to result in loss of NF1 function [43,44,45] (Table 1). Additionally, TK-RIG915 harbours a pathogenic gain-of-function (GOF) mutation in Protein Tyrosine Phosphatase Non-Receptor Type 11 (*PTPN11*) (p. Phe285Ser) [46], which encodes SHP2. SHP2 plays an essential role in the activation of RAS following growth factor receptor activation [47] and GOF mutations have long been associated with childhood leukaemia and other solid tumours [48,49]. While the allelic frequency was high in the PDX (55%), the GOF mutation was rare in the patient tumour (one read out of 50, Table 1), suggesting that a selected subpopulation of tumour cells engrafted and proliferated in mouse brain. Taken together, it appears that activation of the PI3K/AKT and Ras/Raf/MEK/ERK pathways, combined with the loss of the *CDKN2A/B* tumour suppressor locus, drove the growth of this tumour.

### 2.6. RNA Expression Analysis Supports Over-Activation of PI3K/AKT/mTOR and Ras/Raf/MEK/ERK Pathways in TK-RIG915

**Figure 8:**
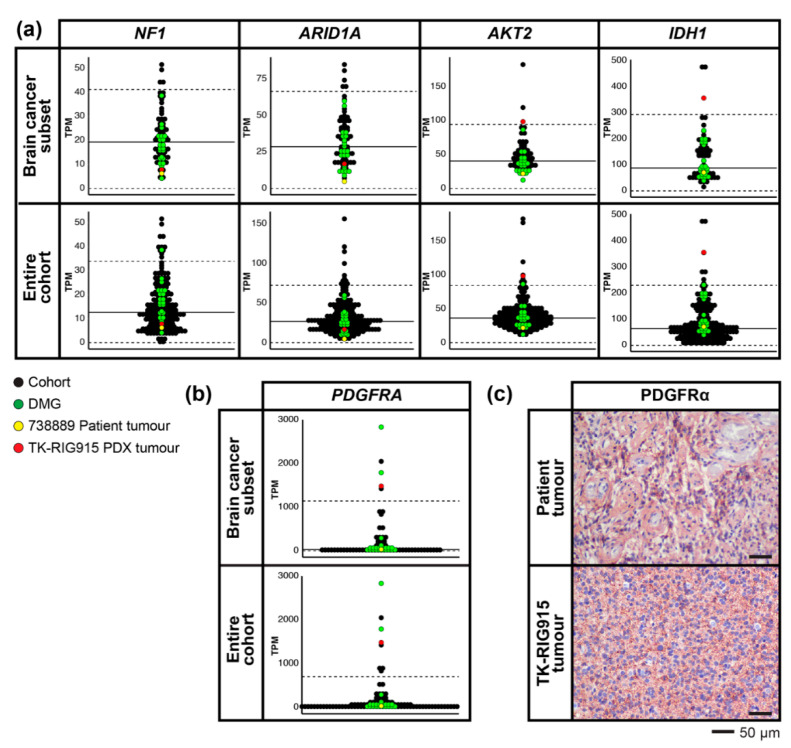
PI3K/AKT/mTOR and Ras/Raf/MEK/ERK pathway genes are over-expressed in TK-RIG915. Gene expression levels for the indicated genes in the patient and matched TK-RIG915 patient-derived xenograft (PDX) tumour tissues (y axis: transcripts per million, TPM). The patient tumour (yellow) and TK-RIG915 (red) are compared with two subsets of a reference cohort containing only high-risk paediatric brain tumours (top panel) or all high-risk paediatric tumours (bottom panel). Diffuse midline gliomas (DMGs) from the reference cohort are indicated in green, all other cancers are black. Solid black line shows the mean TPM of the ZERO cohort, and dashed line shows the TPM values that are two standard deviations away from the mean. (**a**) TK-RIG915 exhibits low *NF1* and *ARID1A* transcript levels, and high *IDH1* and *AKT2* levels. (**b**) High *PDGFRA* transcript levels in TK-RIG915 correlated with (**c**) high PDGFRα protein expression detected by IHC (lower panel). PDGFRα protein expression was also high in the patient tumour (upper panel).

Since WGS suggested PI3K/AKT/mTOR and Ras/Raf/MEK/ERK activation were key mechanisms driving the growth of this tumour, we further explored the RNAseq data to validate these findings. We identified over- or under-expressed genes in the patient tumour and matched PDX relative to either the entire ZERO reference dataset, or just the CNS tumours, using a z-score approach (Methods). *NF1* transcript levels were significantly lower in both the primary and matched TK-RIG915 PDX tumours compared to both reference sets, consistent with other tumours in the reference cohorts that had homozygous deletion of *NF1* (Figure 8a). In addition to low *NF1* expression, we identified significantly lower levels of AT-rich interactive domain 1A (*ARID1A*) transcripts in TK-RIG915 compared to both reference sets (Figure 8a). *ARID1A* is a key subunit of the SWI/SNF chromatin remodelling complex [50] and can indirectly inhibit the PI3K/AKT/mTOR pathway [51,52,53]. As such, loss of this gene may contribute to the overactivation of this pathway already observed in this tumour.

Further evidence that the PI3K/AKT pathway is driving growth of TK-RIG915 is supported by the high levels of *AKT2* and *IDH1* transcripts compared to both reference sets (Figure 8a). AKT2 plays a role in the migration and invasion of glioma cells and expression correlates with the malignancy of gliomas [54]. Similarly, overexpression of wildtype IDH1 has been associated with driving the migration of primary GBM cells via the production of alpha-ketoglutarate, resulting in PI3K/AKT/mTOR pathway activation [55]. Furthermore, tumours with wildtype *IDH1* have a poorer prognosis than WHO-grade matched tumours that harbour *IDH1* mutations [56], suggesting that high expression of the wildtype form of *IDH1* may drive TK-RIG915 tumour progression. High levels of *PDGFRA* RNA expression, which encodes platelet derived growth factor receptor alpha (PDGFRα), compared to both reference sets were also detected in TK-RIG915 (Figure 8b) and confirmed at the protein level by IHC (Figure 8c). Of note, despite low levels of PDGFRα transcripts in the RNAseq data from the patient tumour (Figure 8b), IHC confirmed high PDGFRα protein expression in the tissue (Figure 8c).

### 2.7. IHC Confirms Activated PI3K/AKT/mTOR and Ras/Raf/MEK/ERK Signalling in TK-RIG915

**Figure 9:**
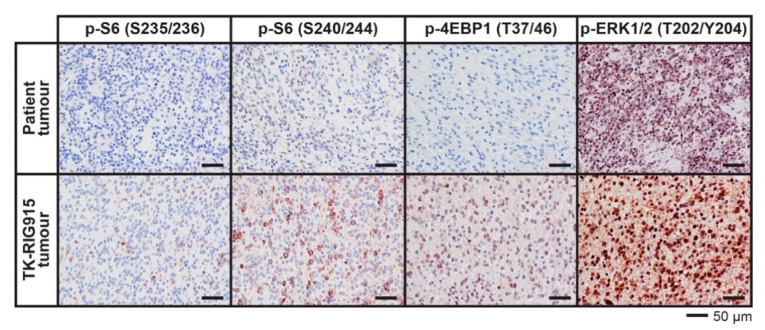
Downstream effectors of the PI3K/AKT/mTOR and Ras/Raf/MEK/ERK pathways are activated in TK-RIG915. Immunohistochemistry for the phosphorylated proteins indicated was performed on the patient tumour and TK-RIG915. The patient-derived xenograft was positive for S6 phosphorylated on residues S240/S244, and to a lesser extent S235/S236, and phosphorylated 4EBP1 T37/46, while only mild staining for phosphorylated S6 S240/244 was seen in the patient tumour. Both tumours were strongly positive for phosphorylated ERK1/2. Scale bar represents 50 µm.

WGS and RNAseq analyses strongly suggested that both the PI3K/AKT/mTOR and Ras/Raf/MEK/ERK pathways were over-active in TK-RIG915. To confirm this, we performed IHC on the tumours to look for activation of downstream effectors of both of these pathways—phosphorylated ribosomal protein S6 and phosphorylated 4EBP1 for the PI3K/AKT/mTOR pathway, and phosphorylated ERK1/2 for the Ras/Raf/MEK/ERK pathway. Both the PDX and the patient tissue were strongly positive for phosphorylated ERK1/2 (Figure 9), confirming Ras/Raf/MEK/ERK pathway activation. Positive staining was also detected for phosphorylated 4EBP1 threonine (T) 37/46 and phospho-S6 serine (S) 240/244, with minimal staining for phospho-S6 (S235/236) in TK-RIG915. The preferential phosphorylation of S6 S240/244 also supports PI3K/AKT/mTOR pathway activation as S240/244 are known targets of S6K1/2 downstream of mTORC1 activation. Limited staining for phospho-4EBP1 or phospho-S6 was observed in the primary patient tumour. Given that the genetic alterations driving the PI3K pathway in TK-RIG915 were generally also observed in the patient tumour, it is possible that the lack of staining was due to technical issues, such as the degradation of the phosphorylated epitopes post-mortem prior to tissue fixation.

## 3. Discussion

Medulloblastoma is the most common malignant brain cancer of childhood. A major component of conventional medulloblastoma therapy is craniospinal irradiation, which is subsequently associated with a risk of radiation-induced secondary neoplasms. Here we describe a case of RIG in a male survivor of medulloblastoma, and the molecular and phenotypic features of the PDX model generated from this case. To our knowledge this is among one of the first PDX models of RIG described, and the first with matched germline DNA available. PDX models are valuable tools for preclinical testing of new therapies, but their full translational potential can only be realised if the underlying genetic landscape of these models is comprehensively understood. Analysis of germline DNA facilitates this by providing confidence in somatic variant calls and allowing identification of any inherited cancer predispositions. In the patient tumour described here, having matched germline DNA was highly valuable for identifying true somatic driver mutations. This tumour was previously reported in Gits et al. [8] where ten mutations were described; however, our analyses revealed that only one of these was a true somatic mutation. This highlights the value of collecting and analysing germline DNA where possible in reporting tumour-associated mutations.

The patient tumour from which this PDX was derived fulfilled the criteria defined by Cahan et al. [31] for being a radiation-induced secondary tumour. Additionally, the features of this tumour in terms of age of diagnosis of the primary medulloblastoma, latency to development of the RIG and overall survival fit well with patterns reported in other cases of RIG following treatment for medulloblastoma [57], and indeed high RNA expression levels of RIG-associated genes were observed [7].

The patient was diagnosed as harbouring a DIPG, according to the WHO-based criteria of the time and based on the MRI characteristics of the tumour. However, our analyses have revealed that the patient tumour and corresponding PDX are molecularly distinct from typical diffuse brain stem gliomas. Specifically, a number of recurrent genetic alterations have been reported for primary DMG including the hallmark H3 K27M mutation, as well as mutations in *TP53, ACVR1* and *PDGFRA*, and amplifications of *EGFR* and *PDGFRA* [3,58,59,60,61]. None of these characteristic genetic alterations were observed in the patient tumour or in TK-RIG915 (although high levels of *PDGFRA* transcript and protein were observed), further supporting that this tumour is distinct from the original diagnosis of primary DIPG.

Donson and colleagues [7] proposed that RIGs closely resemble PAs, with a 39% overlap in highly expressed genes between these two tumour types. TK-RIG915 reflects these similarities, with high expression of *SOX10, ERBB3, PDGFRA, OLIG2, NKX2.2* and *BRINP3* (Figure 5), which have been reported for PAs [7,62,63,64]. It was acknowledged in that report, however, that given the significant differences in WHO grade and clinical outcomes for these patients, these expression profiles may simply indicate that RIGs and PAs share a common precursor cell [7]. Indeed, genetic alterations commonly reported in PAs such as gain of chromosome 7q34 [65] and mutations or fusions involving *BRAF, KRAS, FGFR1* or *NTRK* [66] were not observed in TK-RIG915. Furthermore, while mutations in *NF1* are observed sporadically in PA, these are almost exclusively associated with optic nerve glioma in patients with inherited germline *NF1* mutation, rather than somatically acquired as observed in TK-RIG915 [66,67].

Other than PA, it has been suggested that paediatric RIGs are molecularly similar to adult GBM [8]. Recurrent genetic alterations observed in adult primary GBM include mutations in *PIK3CA* and/or *NF1*, *TERT* promoter alterations, deletion of *CDKN2A/B*, mutation and/or deletion of *PTEN*, mutation and/or amplification of *EGFR* and *PDGFRA*, and overexpression of wildtype *IDH1* [35,68,69,70,71]. In TK-RIG915, we indeed observed several of these alterations, including *PIK3CA* and *NF1* mutations, overexpression of *IDH1* and *CDKN2A/B* deletion (with *CDKN2A/B* loss also reported in the only other published RIG PDXs [15]). That said, there remains a number of hallmark genetic alterations in primary GBM that were not observed in TK-RIG915, and indeed are not observed in the majority of RIGs reported in the literature. For example, most RIGs have normal expression of *EGFR* and *PTEN* and lack *TERT* promoter mutations [7,14,17,18,21,22]. In addition, the majority of published RIGs report mutations in *TP53* [8,14,16,17,18,19,20], which are not normally associated with adult primary GBM although this was also not observed in TK-RIG915.

DNA methylation-based classification has become a widely-accepted method to aid in molecular identification of CNS tumours [29]. Interestingly, the methylation profile of the tumours described here did not match with any known subclass in the MNP2.0 classifier, consistent with the only other reported RIG PDX derived from an autopsy sample [15]. The best match for our patient tumour sample by methylation profiling was “Control tissue, inflammatory tumour microenvironment”. As mentioned, the patient had fungal lesions adjacent to the tumour site, which may indeed have resulted in an inflammatory microenvironment. Furthermore, it is possible that the quality of tissue collected at autopsy, the presence of necrosis and/or the amount of contaminating normal tissue (48% in our case) may affect methylation-based CNS tumour classification of the patient sample [29]. Collectively, our data indicate that whilst some genetic overlap with other tumour types exists, RIGs may be a genetic entity unto themselves, perhaps due in part to sharing a common causative event through radiation exposure. In order to determine if RIGs truly are their own disease entity, extensive molecular characterisation of a large number of RIGs is required to robustly define these tumours in future.

In the case of the RIG we describe here, there were multiple molecular alterations identified that are targetable and may have therapeutic benefit. Firstly, increased activation of the PI3K/AKT/mTOR pathway was identified as a driver of TK-RIG915 pathogenesis. The mutation in *PIK3CA* observed in TK-RIG915 occurs at a cancer-associated hotspot known to upregulate kinase activity [40,41]. Consistent with GBM, the *PIK3CA* mutation in TK-RIG915 was mutually exclusive with *PTEN* mutation/deletion [35]. Other alterations we observed that would be expected to cooperate with PI3K activation included *AKT2* and *PDGFRA* over-expression, both also frequent aberrations observed in glioma [72,73]. Although high transcript levels of these genes were only observed in TK-RIG915, these changes may have been masked in the patient tumour by contaminating normal tissue, rather than these differences being the result of sub-clonal selection in the PDX. Indeed, protein levels of PDGFRα in the patient tumour and TK-RIG915 were concordant. Taken together, these findings suggest inhibitors of the PI3K pathway such as PI3K/mTOR inhibitors or AKT inhibitors may have therapeutic benefit in this PDX [74].

Overexpression of *IDH1* was observed in TK-RIG915 and is also consistent with observations in primary GBM [68]. GBM with wildtype *IDH1* have a worse prognosis than *IDH1*-mutant GBM [56], and it has been recently reported that *IDH1* overexpression, and the subsequent increase in α-ketoglutarate (α-KG), is associated with increased migration of GBM cells and enhanced PI3K/AKT/mTOR pathway activity [55]. Encouragingly, there is evidence to suggest that reducing *IDH1* expression may sensitise GBM cells to radiotherapy, a core clinical treatment for paediatric brain tumours [75].

TK-RIG915 also demonstrated low RNA expression of the SWI/SNF complex component *ARID1A*. Inactivating mutations or reduced *ARID1A* expression is common in multiple cancer types including glioma where it is associated with poorer prognosis [76,77]. There is a significant correlation between loss of *ARID1A* and activating mutations in *PIK3CA* [78], and ARID1A can indirectly inhibit the PI3K pathway via transcriptional regulation of PIK3IP1 (a negative regulator of the PI3K pathway) and Annexin A1 (an activator of AKT) [51,52,53]. As a result, loss of *ARID1A* in TK-RIG915 may contribute to the network of genetic alterations already driving activation of PI3K signalling. Of note, the regulation of PIK3IP1 is dependent on EZH2 methyltransferase and inhibition of EZH2 activity combined with loss of *ARID1A* expression is synthetically lethal [52], highlighting a potential therapeutic vulnerability through the use of EZH2 inhibitors in this tumour.

Lastly, hyperactivation of the Ras/Raf/MEK/ERK pathway was identified in TK-RIG915, where *NF1* loss together with a *PTPN11*/SHP2 GOF mutation was observed with concomitant upregulation of ERK1/2 phosphorylation. In terms of disease initiation, inherited germline mutations in *PTPN11* are associated with Noonan syndrome, where patients have increased risk of cancer development [79]. As it has been shown that *PTPN11* mutations can increase susceptibility to DNA-damage-induced malignancies [80], it is conceivable that this mutation may have been an early somatic event that contributed to the initiation of this RIG. Hyperactivation of the Ras/Raf/MEK/ERK pathway in TK-RIG915 suggests that this tumour may be targetable with MEK inhibitors. NF1-deficient GBMs are sensitive to MEK inhibition, although disease control may require simultaneous PI3K pathway inhibition as has been observed in a subset of NF1-deficient GBMs [81]. Given that both the PI3K and MEK pathways are upregulated in TK-RIG915, this dual inhibition approach may be a promising option in treating this tumour.

**Figure 10:**
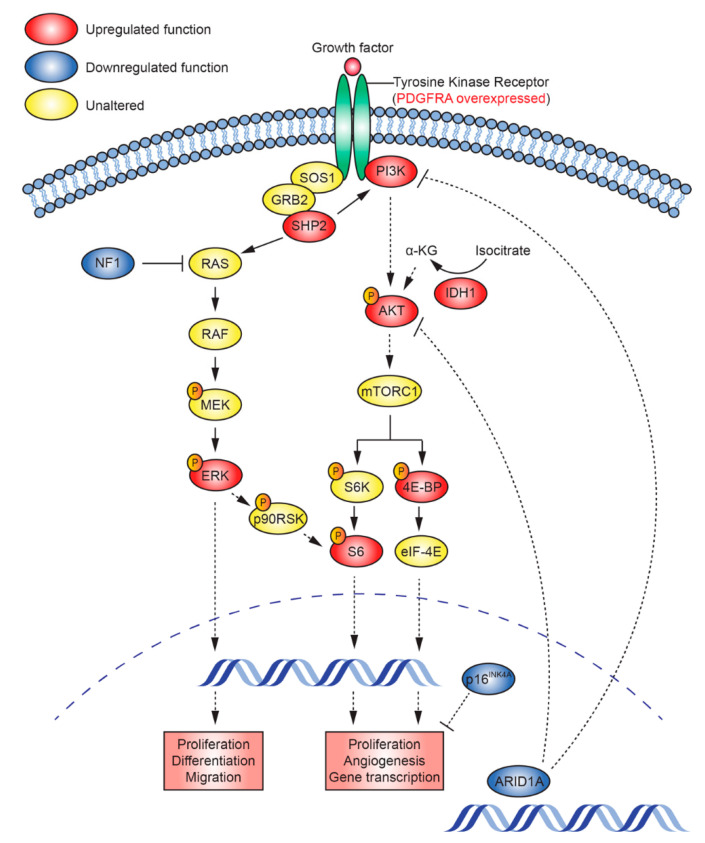
Simplified schematic depicting the cooperating genetic alterations present in TK-RIG915. Shown are a selection of the genetic aberrations observed in this case study and the pathways in which they act (blue indicates genes with downregulated function, red indicates genes with upregulated function or increased expression/phosphorylation). Dashed lines represent indirect effects. 4E-BP—Eukaryotic translation initiation factor 4E-binding protein, AKT—Protein kinase B, ARID1A—AT-Rich Interaction Domain 1A, eIF-4E—Eukaryotic Translation Initiation Factor 4E, ERK—Extracellular signal-regulated kinase, GRB2—Growth Factor Receptor Bound Protein 2, IDH1—Isocitrate dehydrogenase 1, MEK—MAPK/ERK Kinase, mTORC—mammalian target of rapamycin complex 1, NF1—Neurofibromin 1, p16INK4A—cyclin-dependent kinase inhibitor 2A, CDKN2A, p90RSK—90 kDa ribosomal s6 kinase, PDGFRA—platelet-derived growth factor receptor-alpha; PI3K—Phosphoinositide 3-kinase, RAF—Rapidly Accelerated Fibrosarcoma, RAS—Rat sarcoma, S6—Ribosomal protein S6, S6K—Ribosomal protein S6 kinase, SHP2—Src homology region 2, SOS1—SOS Ras/Rac Guanine Nucleotide Exchange Factor 1.

The patient from which TK-RIG915 was derived was offered treatment as part of a clinical trial for DIPG; however, this was ultimately ineffective. Through genomic and transcriptomic analyses we understand now that this tumour was a RIG and is molecularly distinct from typical DIPGs; moreover, this study identified genetic drivers and potential therapeutic targets. The cooperative nature of the genetic alterations driving the PI3K and MEK signalling pathways in TK-RIG915 is summarised in Figure 10. Understanding how these genetic changes drive growth in this tumour provides an opportunity to investigate potential therapeutic targets for RIG. Based on our findings, it is possible that co-administration of inhibitors of MEK, PI3K, mTOR, IDH1, EZH2 or CDK4/6 (to target *CDKN2A* deletion) may have been effective in slowing or reducing tumour progression [52,68,81,82,83,84,85]. Even the use of a different antifungal agent in this patient to treat the fungal brain and lung lesions (for example a dual antifungal agent and mTOR inhibitor such as rapamycin instead of voriconazole) could have potentially had a positive effect. At the time that this patient was diagnosed, biopsies were not routinely performed for brain stem glioma, but a number of clinical trials are demonstrating the feasibility of this approach, and the value that the analyses performed on the biopsy tissue can afford [12,13,86].

This case has uncovered novel insights into radiation-induced brain cancer secondary to medulloblastoma and highlights the importance of enhancing our understanding of these cancers. Most importantly, we have developed and characterised one of the first PDX models of paediatric RIG. The only other reported PDX mouse models of paediatric RIG were derived from a single patient [15], highlighting how rare these models are. In order to facilitate thorough and rigorous preclinical testing of new therapies that these patients may ultimately benefit from, an international effort is required to collect these specimens and expand the number of paediatric RIG PDXs available for research. With the advent of the molecular genetic era, it is abundantly clear that further in-depth characterisation of RIGs is critical in not only building on our understanding of the mechanisms driving disease progression but also in uncovering potential therapeutic vulnerabilities for this universally fatal disease.

## 4. Materials and Methods

### 4.1. Human Samples

The parents/guardians of the patient gave their informed consent before donation of the autopsy tissue for research purposes, and for retrospective research access to relevant medical records and previously obtained pathology samples for the same patient. The study was conducted in accordance with the Declaration of Helsinki, and the protocol was approved by the Ethics Committee of the Child and Adolescent Health Service, Western Australia (HREC: 1769/EP (PRN 0000002372) A Perth Children’s Hospital Oncology Protocol for Collecting and Banking Paediatric Research Specimens; approved 21/08/2003).

### 4.2. Implantation of Patient Autopsy Tumour Cells and In Vivo Serial Transplantation

Animal experiments were approved by the Animal Ethics Committee of the Telethon Kids Institute and performed in accordance with Australia’s Code for the Care and Use of Animals for Scientific Purposes (AEC#242 approved 21/2/2012, AEC#263 approved 1/9/2013, AEC#300 approved 18/4/2016, AEC#362 approved 24/4/2020). Immunodeficient BALB/c nude mice were obtained from the Animal Resources Centre (Murdoch, Western Australia, Australia). Approximately 17 h post-mortem, tumour tissue from the patient (ID 738889) was mechanically dissociated, filtered through a 100 μm cell strainer, and suspended in matrigel (BD Biosciences, San Jose, CA, USA). Cells (4 × 10^6^ per mouse) were implanted into the brains of four 8-week-old mice using a Hamilton syringe. Upon tumour-related morbidity, the brain was bisected sagittally at the implantation site and one half of the brain containing the tumour was kept for histology. The remaining tumour was removed, dissociated and reimplanted into the brains of successive recipients as described above.

### 4.3. Histochemical Staining

Tissue samples were fixed in 4% paraformaldehyde or neutral buffered formalin and embedded in paraffin. Patient medulloblastoma samples were stained with H&E, GFAP (Dako, Santa Clara, CA, USA, Z0344; 1:3000), Ki67 (Ventana, Oro Valley, AZ, USA, 3O-9, neat), β-catenin (Cell Marque, Rocklin, CA, USA, 14, 224M-15, 1:50) and synaptophysin (Ventana, SP11, neat) and patient autopsy RIG tissue was stained with H&E, GFAP, Ki67 and p53 (Dako, DO-7; 1:200) using the Benchmark Ultra Immunostainer using DAB as a substrate (Roche, Basel, Switzerland). Reticulin staining was performed as per Gordon and Sweet’s reticulin staining method. All other tissue sections (5 µm) underwent microwave antigen retrieval in a citrate buffer before immunostaining with the following antibodies and dilutions: Olig2 (Millipore, Burlington, MA, USA, MABN50; 1:200), Nestin (Millipore, MAB5326; 1:200), Vimentin (Cell Signaling, Beverly, MA, USA, 5271; 1:200), Tri-methyl-histone H3 (K27) (Cell Signaling, 9733; 1:200), EGFR (Cell Signaling, 4267; 1:50), PTEN (Cell Signaling, 9559; 1:200), PDGFRα (Cell Signaling, 5241; 1:200), phosphorylated S6 (S235/S236) (Cell Signaling, 2211; 1:400), phosphorylated S6 (S204/S244) (Cell Signaling, 5364; 1:1000), phosphorylated 4EBP1 (T37/T46) (Cell Signaling, 2855; 1:1600), phosphorylated ERK1/2 (T202/Y204) (Cell Signaling, 9101; 1:100). Additionally, mouse PDX tumour samples were stained with GFAP (Sigma Aldrich, St Louis, MO, USA, G3893-2ML; 1:200), Ki67 (Cell Signaling, 9027; 1:400), and p53 (Cell Signaling, 2527: 1:160). Sections were incubated with species-specific biotinylated goat anti-IgG secondary antibodies, followed by detection with an Elite ABC kit and NovaRED peroxidase substrate, then counterstained with Gill’s haematoxylin according to manufacturer’s instructions (Vector Laboratories, Burlingame, CA, USA). H&E staining was performed as per standard protocols using a Leica Autostainer XL.

### 4.4. DNA and RNA Extraction

Genomic germline DNA was prepared from peripheral blood mononuclear cells using a QIAamp DNA Mini Kit (Qiagen, Hilden, North Rhine-Westphalia, Germany, 51304) as per the manufacturer’s instructions for DNA extraction from lymphocytes. Genomic tumour DNA and RNA were prepared from fresh frozen patient and PDX tumour samples using an All Prep DNA/RNA Mini Kit (Qiagen, 80204) as per the manufacturer’s instructions. DNA quality was determined by gel electrophoresis and spectrophotometry (Nanodrop, Thermo Fisher Scientific, Waltham, MA, USA), and quantified using fluorometry (Qubit, Life Technologies, Waltham, MA, USA, Q32851). RNA quality and quantity were determined using the LabChip GX nucleic acid analyser (Perkin Elmer, Waltham, MA, USA) (performed by the Australian Genome Research Facility, Perth, Western Australia, Australia).

### 4.5. Short Tandem Repeat Analysis

DNA from patient tumour and PDX tumours were compared using STR analysis performed at the Genetics Resources Core Facility at Johns Hopkins University (Baltimore, MD, USA) using a PowerPlex 18D kit (Promega, Madison, WI, USA), PCR product electrophoresed on an ABI Prism 3730xl Genetic Analyzer and data analysed using GeneMapper v4.0 software (Applied Biosystems, Foster City, CA, USA) as per their standard protocols.

### 4.6. Methylation Array

Genomic DNA (500–1000 ng) was treated with sodium bisulphite using the EZ DNA methylation kit (Zymo Research, Orange, CA, USA) and bisulphite conversion was confirmed by methylation specific PCR. Quantification of DNA methylation was performed at the Australian Genome Research Facility (Melbourne, Victoria, Australia) using the Human Methylation EPIC (EPIC) BeadChip run on an Illumina iScan System using the manufacturer’s standard protocol (Illumina, San Diego, CA, USA). Raw idat files were uploaded to an online DNA methylation-based classification of CNS tumours platform (www.molecularneuropathology.org, version 11b4 v2.1) [29] and basic copy number variant profiles from methylation array data analysed using the output generated from this classifier.

### 4.7. Whole Genome Sequencing

WGS data obtained from the patient germline and tumour DNA samples were analysed as reported in [30]. For the PDX tumour sample, an additional step to remove mouse reads using BBSplit ver 11 June 2018 [87] was done prior to the previously described method. Default parameters were used except for ambiguous2 that was set to ‘toss‘ in order to conservatively exclude ambiguously mapped reads to either the mouse or human reference genomes. WGS data generated by this study are available from the European Genome-phenome Archive under accession number EGAS00001004709.

### 4.8. RNA Sequencing, Clustering Analysis and Expression Profiling

RNAseq analysis and expression profiling was performed as reported in [30]. Clustering analysis was performed using R package Rtsne by combining the transcripts per million (TPM) values from the ZERO cohort [30] with the primary tumour and PDX model TPM values. RIG signature analysis was performed on the genes identified in [7] where the cohort median and mean values were computed and either the patient tumour TPM or PDX model TPM of the genes specified were compared to the cohort mean and median. RNAseq data generated by this study are available from the European Genome-phenome Archive under accession number EGAS00001004709.

## 5. Conclusions

To date, the genetic and molecular alterations underpinning RIG have not been well-characterised. We have added to this limited body of knowledge through extensive characterisation of one of the first PDX models of paediatric RIG. The case report we describe here and TK-RIG915 were molecularly distinct from typical paediatric brain stem glioma and instead showed some genetic overlap with adult primary GBM, concordant with other reports. Whilst some similarities between these tumour types exists, there remain key differences between patterns of genomic and transcriptomic alterations between adult primary GBM and TK-RIG915, and indeed the majority of RIGs previously reported, suggesting that perhaps RIGs may form their own CNS tumour subtype. Extensive characterisation of an increased number of RIGs is crucial to definitively address this possibility and increase our understanding of these tumours. Interrogation of the genetic alterations present in this case revealed a number of potential therapeutic targets that may be effective against this disease including inhibitors of MEK, PI3K, AKT, mTOR, IDH1, EZH2 or CDK4/6. Through the development of a rare RIG PDX, we have created an important tool to help facilitate both drug discovery and novel therapeutic preclinical testing for this highly aggressive and currently fatal disease.

## Figures and Tables

**Table 1:** TK-RIG915 harbours pathogenic mutations in *PIK3CA, NF1* and *PTPN11*. Allelic frequencies for both TK-RIG915 and the matched patient tumour calculated from whole genome sequencing data are shown.

Gene	Coding Mutation	Impact	Likely Effect	Amino Acid Alteration	Allelic Frequency	
					Patient Tumour	TK-RIG915
*PIK3CA*	c.3140A > T	Missense	Activating	p. His1047Leu	24%	43%
*NF1*	c.3367G > T	Stop-gain	Inactivating	p.Glu1123Ter	32%	45%
*NF1*	c.233dupA	Frameshift	Inactivating	p.Asn78LysfsTer29	20%	62%
*PTPN11*	c.854T > C	Missense	Gain of function	p. Phe285Ser	2%	55%

## Supplementary Materials

The following are available online at https://www.mdpi.com/2072-6694/12/10/2937/s1. Table S1: Penetrance and median survival rates of the TK-RIG915 patient-derived xenograft model, Table S2: Short Tandem Repeat (STR) analysis demonstrated that the patient-derived xenograft (PDX) tumours were derived from the patient tumour, Table S3: The majority of the mutations reported in Gits et al. [8] were found in the patient’s germline DNA, Figure S1: Enlarged CIRCOS plot for patient tumour 738889, Figure S2: Enlarged CIRCOS plot for PDX TK-RIG915.
