# Supplementary material for: A Novel Orthotopic Patient-Derived Xenograft Model of Radiation-Induced Glioma Following Medulloblastoma
Source: Cancers (Basel). 2020 Oct 12;12(10):2937. doi: 10.3390/cancers12102937 (PMC7600047; doi:10.3390/cancers12102937)
Supplement: Supplementary file 1 [file cancers-12-02937-s001.pdf]

cancers

Supplementary Materials

A Novel Orthotopic Patient-Derived Xenograft Model of Radiation-Induced Glioma Following Medulloblastoma

Jacqueline P. Whitehouse, Meegan Howlett, Hilary Hii, Chelsea Mayoh, Marie Wong, Paulette Barahona, Pamela Ajuyah, Christine L. White, Molly K. Buntine, Jason M. Dyke, Sharon Lee, Santosh Valvi, Jason Stanley, Clara Andradas, Brooke Carline, Mani Kuchibhotla, Paul G. Ekert, Mark J. Cowley, Nicholas G. Gottardo and Raelene Endersby

Table S1. Penetrance and median survival rates of the TK-RIG915 patient-derived xenograft model. The number of mice implanted in each generation and the number of mice euthanised due to tumour-related and non-tumour-related reasons are shown, as well as how many days since tumour implant that euthanasia occurred for each mouse. Non-tumour related reasons included rectal prolapse or serious skin conditions, which are complications common to the immunocompromised Balb/c nude strain of mouse.

Implant generation	Median survival (days)	Total number of mice implanted	Number euthanised due to tumour (days since implant)	Number euthanised due to non-tumour related reasons (days since implant)	Penetrance
Patient tumour	170	4	4 (147, 147, 194, 194)	0	100%
Secondary implant	78	11	8 (55, 55, 57, 78, 84, 129, 148, 172)	3 (27, 64, 64)	73%
Tertiary implant	97	17	9 (67, 70, 70, 86, 90, 97, 100, 100, 105)	8 (52, 52, 72, 93, 122, 230, 273, 273)	53%

Table S2. Short Tandem Repeat (STR) analysis demonstrated that the patient-derived xenograft (PDX) tumours were derived from the patient tumour. Results from STR analysis of patient tumour DNA and DNA obtained from PDX tumours from mice in the secondary and tertiary implant generations for each locus shown.

Loci	Patient Tumour DNA	DNA from Secondary Implant PDX Tumour	DNA from Tertiary Implant PDX Tumour
AMEL	X,Y	X,Y	X,Y
CSF1PO	11	11	11
D13S317	9,15	9	9
D16S539	11,13	11,13	11,13
D18S51	14,19	14,19	14,19
D19S433	12,16	12,17	12,17
D21S11	28,29	28,29	28,29
D2S1338	17,20	17,20	17,20
D3S1358	15,17	15,17	15,17
D5S818	9,12	9,12	9,12
D7S820	8,10	8,10	8,10
D8S1179	13,16	13,16	13,16
FGA	20,22	20,22	20,22
Penta D	10,14	10,14	10,14
Penta E	5,15	5,15	5,15
TH01	8,9	8,9	8,9
TPOX	8	8	8
vWA	16,19	16,19	16,19

Table S3. The majority of the mutations reported in Gits *et al* [8] were found in the patient's germline DNA. One mutation (PIK3CA H1047L) was a confirmed somatic mutation and was detected in tumour DNA only, seven mutations were found in germline DNA, and two mutations (PRSS3 K186E and KMT2C Q755*) were not found in germline or tumour DNA in our analysis.

Gene	Amino acid change	Allelic fraction (%) reported in [8]	Present in germline DNA
EPHA8	p.R884H	85	Yes
GRK4	p.R26C	57	Yes
FLG	p.R971H	47	Yes
FLG	p.E2074Q	45	Yes
BAZ2A	p.R1793W	45	Yes
PIK3CA	p.H1047L	38	No
EGFR	p.R791R	38	Yes
CRTC3	p.R70Q	35	Yes
PRSS3	p.K186E	16	No
KMT2C	p.Q755*	14	No

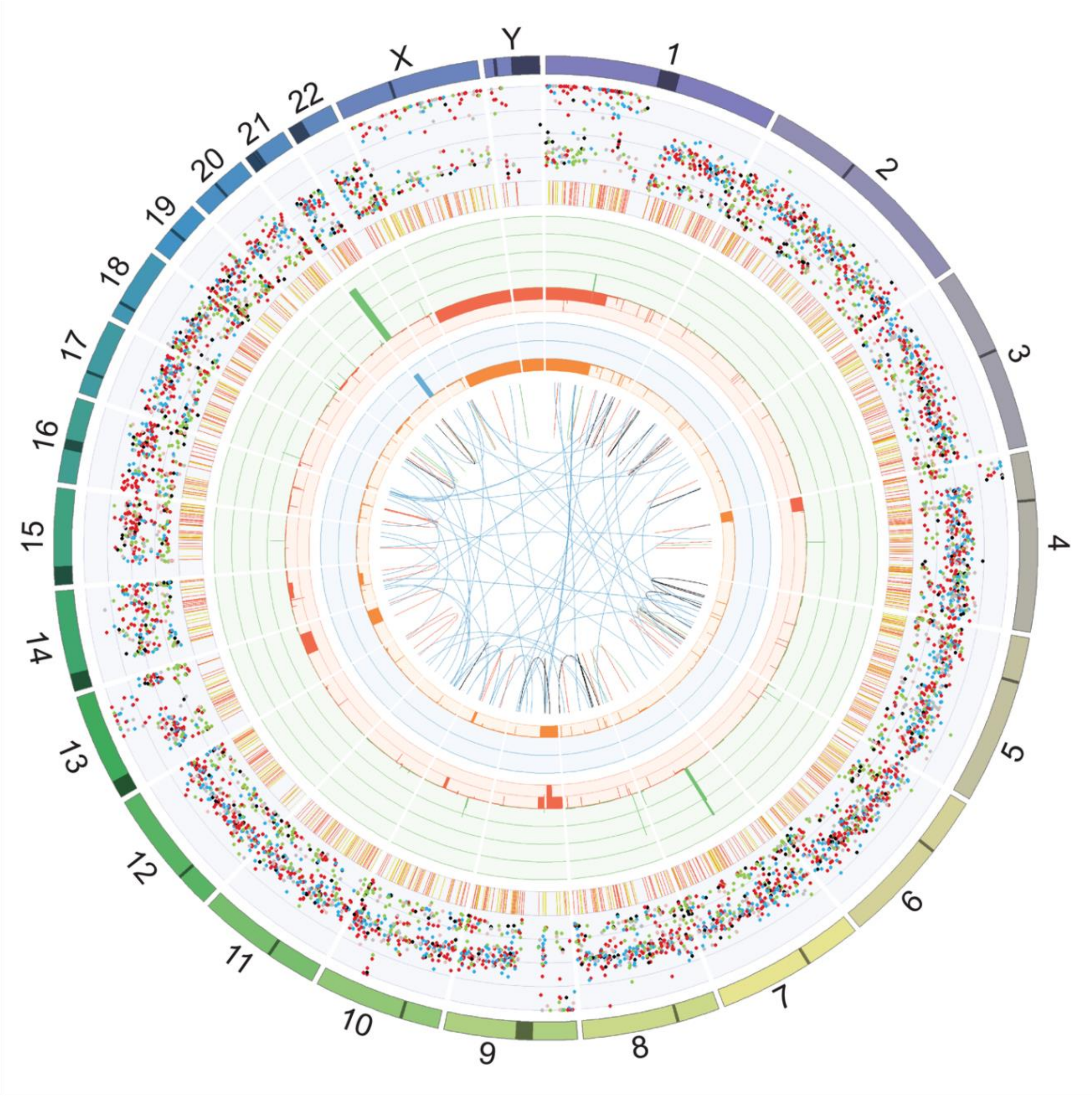

Figure S1. Enlarged CIRCOS plots for patient tumour 738889 from Figure 6C. Key to the image is as described in the legend of Figure 6.

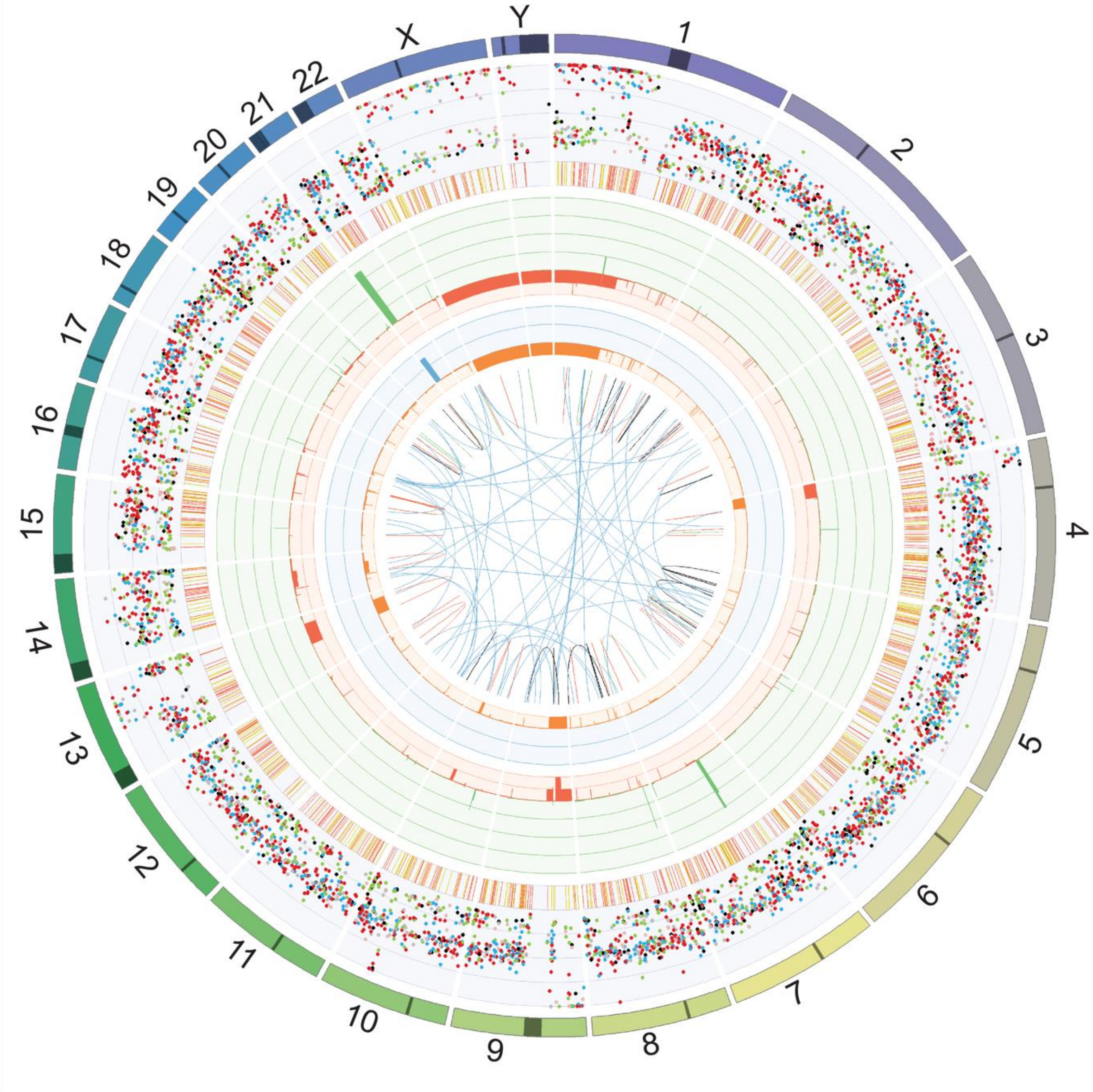

Figure S2. Enlarged CIRCOS plot for PDX TK-RIG915 from Figure 6D. Key to the image is as described in the legend of Figure 6.

© 2020 by the authors. Licensee MDPI, Basel, Switzerland. This article is an open access article distributed under the terms and conditions of the Creative Commons Attribution (CC BY) license (http://creativecommons.org/licenses/by/4.0/).
